# l-theanine: From tea leaf to trending supplement – does the science match the hype for brain health and relaxation?

**DOI:** 10.1016/j.nutres.2024.12.008

**Published:** 2025-01-02

**Authors:** Roderick Dashwood, Francesco Visioli

**Affiliations:** a Department of Translational Medical Sciences, Texas A&M University Naresh K. Vashisht College of Medicine, and Center for Epigenetics & Disease Prevention, Texas A&M Health, Houston, TX, USA; b Department of Molecular Medicine, University of Padova, Padova, Italy; c IMDEA-Food, Madrid, Spain

**Keywords:** Brain health, Clinical trials, Cognitive function, Green tea, Relaxation, l-theanine

## Abstract

l-Theanine is a unique non-protein amino acid found abundantly in tea leaves. Interest in its potential use as a dietary supplement has surged recently, especially claims related to promoting relaxation and cognitive enhancement. This review surveys the chemistry, metabolism, and purported biological activities of l-theanine. It is well absorbed from the intestine and can cross the blood-brain barrier. Some studies suggest l-theanine may increase alpha waves in the brain associated with relaxation and selective attention, reduce stress and anxiety, and improve sleep quality, though findings are often inconsistent. Potential neuroprotective and anti-seizure effects have also been reported in animal models. When combined with caffeine, l-theanine may improve cognitive performance, alertness and focus. However, the evidence supporting many health claims remains limited, especially the lack of rigorous human clinical trials. While l-theanine exhibits a good safety profile based on toxicology studies, caution is warranted regarding the purported health benefits, until stronger scientific substantiation emerges. Overall, the mechanisms of action and therapeutic potential of l-theanine require further investigation, given the current interest and increasing popularity of this nutraceutical supplement marketed for brain health and relaxation. In the absence of well-designed and carefully controlled human clinical trials, we would urge caution in the use of l-theanine supplements at pharmacologic doses by the wider population, and believe that the science does not yet match the hype behind this trending supplement for brain health and relaxation.

## 1. Introduction

Green tea consumption has been associated with enhanced longevity, owing to lower cardiovascular mortality and incidence of cancer [1–5]. Although cause-and-effect often has been difficult to establish, with “inconclusive” and “inconsistent” findings reported in the literature [1], green tea and its constituents remain the subject of active scientific pursuit. One notable example is epigallocatechin-3-gallate (EGCG) and other structurally-related polyphenolic tea catechins, for which there is ample preclinical and cell-based mechanistic research, plus ongoing efforts at human clinical translation [6–8].

One other green tea constituent that is receiving increased attention is l-theanine, based on the associated psychoactive properties and other purported health benefits [9]. Interest in the biological activities of l-theanine currently is gaining great traction because of its use in health supplements, with claims of central nervous system benefits, and marketed accordingly. Whether there is adequate mechanistic evidence or supporting information for such health claims remains open to debate. The published literature on l-theanine is inconsistent, sometimes lacking appropriate rigor and reproducibility, especially in the context of the evidence from human clinical trials. Hence, we believe it is timely and appropriate to review this topic, given the ongoing “hype” regarding l-theanine in popular health supplements targeted towards mood and relaxation. This approach aligns with a recent initiative of the International Union of Basic and Clinical Pharmacology (IUPHAR) calling for rigorous evidence regarding the pharmacology of natural products [10–12].

We briefly survey the chemistry of l-theanine and discuss its established and putative biological activities. Prior reviews on the subject of l-theanine considered such aspects as cognitive outcomes [9], cancer etiology [13], immunity [14], signaling cascades [15], food applications [9], and metabolism/transport in tea plants [16]. Caution must be exercised in separating apparent “proof” or “fact” from anecdotal evidence, for any given mechanism of action, especially in the context of human subject intake via supplementation.

## 2. Background

l-(γ-l-glutamylethylamide) is a non-nutritive factor that came to public attention as a nootropic agent with potential sedative and mood relaxing effects. It is an amino acid that shares structural similarities with l-glutamate and l-glutamine, but is not involved in the production of enzymes or structural proteins with essential roles in cellular biochemistry. l-theanine is most often associated with the leaves of *Camellia sinensis*, the source of green tea catechins [17], and in other tea varieties following partial or complete oxidation steps during commercial production [18]. White, green, oolong, and black teas typically contain 0.9 to 3.1 percent of the dry weight of the leaves, and thus 5.8 to 32 mg per 200 ml of brewed tea (3 g of dried tea leaves) [19,20]. Remarkably, l-theanine can comprise up to 50 percent of the total amino acids in tea. However, levels also vary depending on temperature, season, cultivar, growth period and other conditions (reviewed by Li et al. [9]). Additional sources of l-theanine include *C. japonica* and *C. sasanqua* [21], as well as the edible mushroom *Xerocomus badius* [22].

The tea content of l-theanine varies markedly according to the cultivar, growth period, early versus late buds or leaves [9], seasonality, and brewing method [9,23]. An investigation of 37 commercial varieties of tea reported an average content of 6.56 mg/g [24]. Another study [25] noted that, contrary to popular wisdom, a standard 200 ml cup of black tea contained the most l-theanine (24.2 ± 5.7 mg), while an equivalent cup of green tea contained the least (7.9 ± 3.8 mg) [25]. Alcázar et al. [26] evaluated the content of commercially available teas and reported that l-theanine was abundant and accounted for more than 50% of the amino acid fraction of the tea samples. Concentrations ranged from 0.07 mg/g of pu-erh tea to 33.37 mg/g of white tea, with wide sample variation noted among the tea varieties. Similar observations were reported by Syu et al. [27] and Thippeswami et al. [28]. Five mg/g dry leaves were the average content of commercially available teas, but the human intake via tea was variable and inconsistent – even for frequent consumption. Thus, at the present time, the L-theanine content of brewed tea appears to be non-standardizable and varies markedly according to cultivar and brewing method. This no doubt provided an impetus for the development of l-theanine supplements, for which QA/QC might be better controlled, especially with a view to future human interventional trials.

## 3. Structure

**Fig. 1 –:**
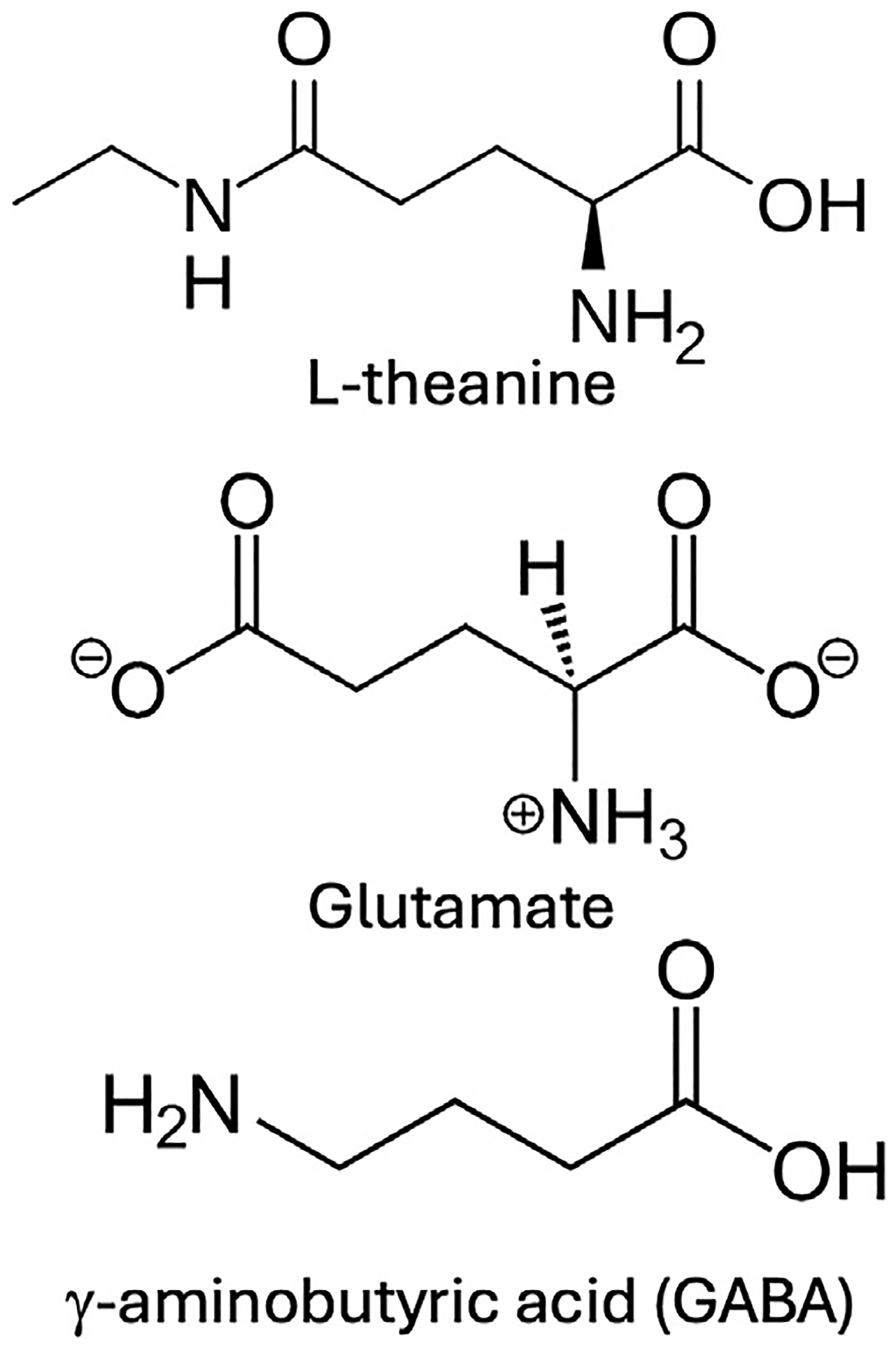
l-Theanine exhibits structural similarities to the neurotransmitters glutamate and GABA.

l-Theanine exhibits structural similarities to the neurotransmitters glutamate and *γ*-aminobutyric acid (GABA). Thus, substitution of the carboxylic acid end of glutamic acid, furthest from the amino group, with an amino moiety followed by an ethyl group produces l-theanine [29]. The addition of an amide group to glutamate produces glutamine, and thus l-theanine also can be considered a structural analog of glutamine (Fig. 1).

## 4. Taste

l-Theanine is reportedly capable of altering taste perception by reducing bitter flavors in chocolate, caffeine, grapefruit and other foods, and is responsible for the “umami” taste of green tea [30]. Being synergistic with the molecule that is employed commonly in umami taste studies, inosine 5′-monophosphate [30], L-theanine appears to be the only molecule in green tea that stimulates the pleasant umami sensation. Oxidation processes – referred to erroneously in the tea industry as “fermentation”– can reduce the overall L-theanine content, whereas drying (40–55 °C for 7–8 hours) appears to increase the overall percentage of l-theanine by dry weight. Younger plants typically have a higher l-theanine content than older plants [31]. Thus, the more expensive and better-quality green tea varieties derived from early harvest of “two leaves and a bud” typically are associated with higher levels of l-theanine.

## 5. Metabolism

In the rat, l-theanine was metabolized renally through the phosphate-independent variant of the enzyme glutaminase [9]. The phosphate-dependent version of the enzyme does not appear to play major roles in metabolism. The metabolism is similar to that of glutamine. l-Theanine is well absorbed from the intestine; in rats, at 2 hours after a single oral dose, plasma l-theanine was significantly increased, and the levels reached 1.24, 3.11, and 4.30 μmol/mL when the rats received 100, 200, and 400 mg of l-theanine, respectively [32]. In mice, the serum concentration of l-theanine peaked 15 minutes after oral administration and was followed by rapid elimination without affecting circulating endogenous amino acids [33]. In general, concentrations attainable in the brain of rodent models are equivocal and require further investigation.

In human subjects, a randomized crossover trial compared the kinetics of L-theanine and its metabolites, glutamic acid and ethylamine, in healthy participants. As discussed further, below, peak plasma concentrations of L-theanine were in the range 24.3 to 26.5 μM following ingestion of l-theanine via capsules and green tea [34]. A human trial compared the bioavailability of l-theanine after consumption of equivalent doses (100 mg) in capsule form or contained in green tea. The data revealed a plasma peak 0.8 h after ingestion, reaching a concentration of about 25 μM [34].

## 6. Longevity

l-Theanine supplementation in *Caenorhabditis elegans* at concentrations of 0.1 to 10 μM prolonged average lifespan by 3.6% and increased maximum lifespan by 4.4% [35]. However, outcomes were not concentration-dependent, with 100 nM l-theanine being the most effective [35]. These properties are certainly not unique to l-theanine, and have been widely reported for numerous other dietary phytochemicals, including EGCG, epicatechin, resveratrol, quercetin, sulforaphane, and curcumin [36,37], with the usual caveats for limited evidence in human subjects [38–40].

## 7. Neurology

Oral intake and parenteral administration via intramuscular injection increased the concentrations of l-theanine in the brain [41,42]. Crossing the blood-brain barrier was mediated by the leucine transport system regulating neutral amino acid transport [43,44]. Following oral intake, l-theanine reached the brain within one hour and its concentration remained elevated for up to 5 hours, after which the levels decreased and disappeared by 24 hours [42]. Concentrations reached in the brain via oral dosing of 4,000 mg/kg body weight were about 2 μM/g [42]; serum concentrations peaked above 12.5 μM within one hour and declined to brain-like concentrations after 16 hours [42]. After oral intake, an increase in l-theanine concentration was detected in the hippocampus among other brain areas [45].

l-Theanine associates with several receptors in the brain, particularly those related to glutamate and cannabinoid signaling. In particular, l-theanine was identified as a competitive inhibitor of the cannabinoid receptor 1 (CB1). This interaction suggests a role for l-theanine in modulating glutamine metabolism and immune function through the corresponding receptor signaling pathways [46]. l-Theanine also interacts with various glutamate receptors, including kainate and *α*-amino-3-hydroxy-5-methyl-4-isoxazolepropionic acid (AMPA) receptors, with IC_50_ values reported as 80- to 30,000-fold less effective than that of L-glutamic acid [47]. This indicates that while it can bind to the receptors, l-theanine does so with significantly lower affinity than glutamate.

Also, l-theanine has been characterized as a partial agonist at the glycine binding site on N-methyl-D-aspartate (NMDA) receptors, suggesting a complex role in modulating excitatory neurotransmission [48]. As regards GABA receptors, although not directly quantified in terms of IC_50_ or EC_50_, l-theanine is noted for its ability to enhance GABAergic activity, which may contribute to its purported calming effects [49]. These interactions provide biochemical grounds for l-theanine as a potential modulator of neurotransmitter systems in the brain, particularly in relation to stress and cognitive function.

Some reports noted that l-theanine intake at standard supplement dosages (50–250 mg) increased alpha waves in healthy people and promoted a state of psychophysical relaxation. This relaxation was observed only in people with slightly higher than average basal levels of anxiety [50,51] or during periods of stress. However, such findings are considered to be controversial or inconsistent, because the literature reports both positive [41] and negative effects on relaxation by l-theanine [52]. Changes in alpha waves also occurred during visuospatial reactivity experiments [53], 30 to 60 minutes after ingestion of l-theanine [19,50], and were exhibited by alpha-1 (8–10 Hz) but not alpha-2 waves (11–13Hz) [19].

It is known that alpha waves are associated with a relaxed state [54]; a similar behavioral change has been noted to occur in parallel with alpha wave production after l-theanine ingestion [53]. In addition to relaxation, the increase in alpha waves is associated with mechanisms of selective attention [55] and mental stimulation/readiness [56]. The effects of l-theanine on alpha waves would appear to be divergent but can be explained by the induction of a state of “calm attention.” Such changes in brainwaves would represent evidence, albeit indirect, that l-theanine indeed possesses relaxing properties while promoting attention. One study reported an increase in theta waves, normally associated with deep sleep, with a combined supplement of l-theanine (60 mg) and green tea extract (360 mg) taken 3 times daily for 16 weeks [57]. No such evidence currently exists for l-theanine alone.

Perhaps the most comprehensive investigation to date in healthy human volunteers [58,59] involved administering a green tea extract that was decaffeinated and enriched in l-theanine. The results showed a general attenuation of delta waves during the first hour after administration. In a reading test, there was an increase in delta and theta waves in the frontal part of the brain, which would suggest increased intellectual performance. Increases in beta1 waves beginning in the second hour, on the other hand, indicated a relaxed state. An increase in alpha wave frequency indicative of relaxation without drowsiness also was observed, for example, in meditation practitioners who administered l-theanine to volunteers in amounts consistent with tea consumption [19].

It is noteworthy that a pilot study with eight human volunteers administered green tea or black tea recorded a change in brain electrical activity. Within an hour of tea intake, electroencephalograms of the volunteers showed increased alpha, beta, and theta waves, with large inter-individual variations and difficult physiological interpretation [60]. In terms of neuroanatomical regionality, one report noted an increase of attention-related anticipatory alpha waves over the right parieto-occipital scalp, suggesting that l-theanine might exert area-specific effects on attention circuitry in the brain [61], in agreement with previous data from Foxe et al. [62]. It must be reiterated that, to date, the proposed state of ‘calm alertness’ can be obtained only via the use of pharmacologic doses of l-theanine. Thus, collectively, the results of electrophysiology studies and data obtained in preclinical models and humans suggest that certain components of green tea, such as l-theanine and catechins, may increase intellectual acuity while inducing a state of relaxation.

## 8. Sedation

Some studies measuring brain alpha wave production also noted that participants independently reported a more relaxed state [50]. l-Theanine administered via intramuscular injection to rats at a dose of 5 to 10 mM/kg body weight increased hexobarbital-induced sleep time by 11–21%, but not in a dose-dependent manner [41]. Attention Deficit/Hyperactivity Disorder (ADHD) tends to be associated with hyperactivity-related symptoms, such as restless legs syndrome or disturbed sleep [63]. Subjects 8–12 years of age with ADHD were treated with 200 mg of l-theanine twice daily for 6 weeks. Sleep quality appeared to be improved by reducing motility during sleep [64]. Sleep latency is defined as the time required to fall asleep, whereas sleep duration is the time elapsed between going to bed and waking in the morning. These parameters were not affected by l-theanine intake [64]. Furthermore, doses of l-theanine used to promote relaxation did not have hypnotic or sedative side effects [65]. Thus, it appears that l-theanine acts on sleep through anxiolytic and not hypnotic effects [65].

## 9. Memory

One investigation examined a supplement called LGNC-07, containing 360 mg green tea extract and 60 mg l-theanine, given 3 times a day for 16 weeks to people with mild cognitive impairment, based on Mini-Mental State Examination (MMSE) scores. Supplementation was associated with better performance on a recognition test and improved recall scores, with no apparent effects on verbal and visuospatial memory using the Rey-Kim test [57].

## 10. Seizures

Effects on seizure induction and resolution were complex based on studies in animal models treated intraperitoneally with l-theanine, and thus not representative of normal human consumption [66]. In other reports, oral intake of l-theanine at 4% in the drinking water was effective against pilocarpine-induced seizures in the rat, but augmented seizures stimulated by pentylentetrazole [67]. The authors suggested the use of l-theanine in the treatment of limbic but not generalized seizures, and hypothesized that the mechanism was related to reduced GABA concentrations in the frontal cortex [67]. An enhancement of seizures by pentylentetrazole, a GABA antagonist [68] also known as Corazol/Cardiazol, was noted by other authors with both green tea and black tea containing L-theanine [69].

## 11. Anxiety

A comparative investigation between 200 mg L-theanine and 1 mg of alprazolam, as a control, on anticipatory anxiety found that while l-theanine promoted relaxation, both L-theanine and alprazolam did not significantly reduce symptoms in the anticipatory anxiety model [52]. Another report also documented no differences between l-theanine and placebo at this dose [51]. In studies assessing relaxation [51,52] or attention/reaction time [51], only people with high baseline anxiety noticed benefits associated with relaxation, whereas those who were not anxious failed to derive benefits beyond the placebo. A systematic review and network meta-analysis of herbal treatments reported that L-theanine did not outperform placebo, in contrast to silexan and other standard treatments for anxiety disorders [70].

## 12. Attention

In people with mild cognitive impairment, a supplement containing both green tea extract (360 mg) and l-theanine (60 mg) administered for 16 weeks improved selective attention assessed by a Stroop test [57]. An improvement in attention was noted in otherwise healthy people with high baseline anxiety, with no noticeable effect on those with lower anxiety scores at baseline [51].

## 13. Stress

Rats administered 0.3 percent l-theanine in drinking water had lower blood levels of corticosterone, both at rest [45,71] and after a stress test [45,72], compared with the control group. In hippocampal CA1 cells, l-theanine caused a shift from NMDA-dependent long-term potentiation (LTP) to NMDA-independent potentiation [71], slowing stress-induced memory impairment [45,72]. Increases in corticosterone [73] and stress [74] can suppress LTP and memory processing in the hippocampus; a reduction in corticosterone concentrations is thought to underlie the protective effects of l-theanine in this context [45,72]. Supplementation with 200 mg of l-theanine before an arithmetic test involving mental calculations reduced perceived stress following administration of the task and reduced salivary IgA concentrations, a stress biomarker, by about half at the end of the task [41].

## 14. Blood flow

Green and black teas both stimulate vascular reactivity [75,76] and may increase nitric oxide production [75,77]. l-Theanine appears to promote nitric oxide formation via phosphorylation on Ser 1177 of the endothelial nitric oxide synthase (eNOS) enzyme variant, with concentration-dependent effects between 0.01–1 μM [78]. The phosphorylation of eNOS and subsequent endothelial relaxation was PI3K/ERK dependent and independent of Akt [78]. It is conceivable that part of the cerebral effects attributed to l-theanine are mediated by improved vascular flow and lower inflammation [79], although no definite evidence exists to date.

## 15. Nutrient interactions

### 15.1. Caffeine

The rationale for co-ingesting or co-administering caffeine and l-theanine for nootropic effects is that the former is absorbed more rapidly than the latter, potentially sustaining alertness [80]. Indeed, there is some limited evidence of moderate effects sizes of combined caffeine and l-theanine 2 hours post administration on some outcome measures of alertness [80], which might stimulate marketing of nutraceuticals that combine the 2 molecules. Cognitive testing in healthy adults revealed that 100 mg of l-theanine plus 50 mg of caffeine augmented accuracy and attention parameters more effectively than caffeine alone [81]. Further dose-finding improved sustained attention and perception of fatigue [82]. l-Theanine and caffeine did not reduce the number of errors in a sustained attention task when comparing the combination therapy with either treatment alone [83].

Caffeine ingested at 150 mg orally improved fatigue perception, rapid visual information processing (RVIP), and reaction time. The addition of 250 mg l-theanine preserved these benefits while enhancing alertness, improving reaction time, and reducing the incidence of headache – which was increased in the caffeine control group [84]. Reaction time as well as the ability to switch between tasks was improved by combination therapy at lower doses, i.e., 50 mg caffeine plus 100 mg l-theanine [81]. Improved attention and fewer effects of distracting stimuli were also reported [81].

At least one study reported that the increase in attention assessed by tasks requiring switching between tests, with some distracting stimuli included by the researchers, occurred independently of the observed perception of attention [85]. A more recent investigation conducted in 12 volunteers who were regular or occasional caffeine users compared the effects of caffeine (75 mg), l-theanine (50 mg), or a combination of both agents on brain flow and parameters of emotionality and reactivity [85]. The results showed that the combination of l-theanine and caffeine circumvented the vasoconstrictor effects of the latter, without the former having vascular effects. These data reinforced prior work [86,47] on beneficial changes in blood pressure when l-theanine was combined with caffeine [87]. No noteworthy effects on behavior were recorded, although the caffeine plus l-theanine combination slightly reduced the speed with which subjects performed mathematical calculations compared with caffeine alone.

### 15.2. Green tea

In the Caco-2 intestinal transport assay, l-theanine had moderate bioavailability when cells were incubated with green tea [88]. Through passive diffusion, l-theanine was generally well absorbed at concentrations above 4 mM, which are excreted from intestinal cells into the circulation. Below this concentration, bi-directional absorption was noted, namely apical to basolateral as well as basolateral to apical. An incubation study with green tea noted that while the rate of absorption was only hindered by 35%, the rate of efflux was increased dramatically; thus, high starting concentrations can proportionally reduce the relative rate of absorption [88]. Although no causal link was demonstrated, it was postulated that small amounts of d-theanine in green tea (2.2–4.7% of the total content [89]) could compete with l-theanine [90]; however, other authors have questioned this possibility [88].

### 15.3. Glutamine

Glutamine appears to share the same intestinal transporter as l-theanine, a sodium-coupled villus transporter, except with much higher affinity [90]. Absorption of glutamine and l-theanine [88] across the intestinal membrane also occurs via passive diffusion, suggesting similar absorption kinetics. A study noting lower absorption of l-theanine administered with green tea compared to pure l-theanine suggested that glutamine in green tea might be responsible for this effect, but this hypothesis remains unsubstantiated [88]. Tannic acid, a major constituent of green tea, also may inhibit the mitochondrial glutamate transporter [91], but has not been studied on the intestinal glutamine transporter.

## 16. Safety and toxicology

### 16.1. General

Tea has been consumed throughout the world for thousands of years, with no adverse effects in the population at-large. In terms of potential use as a supplement, available data showed that oral ingestion of 99% l-theanine produced no toxicity in rats at 6500 mg/kg for 2 weeks or 2000 mg/kg for 28 days [65]. Five percent of the diet as l-theanine for 78 weeks produced no toxic effects or carcinogenicity. A 13-week toxicity test in rats, conducted according to OECD criteria, established a no observed adverse effect level (NOAEL) limit of 4000 mg/kg body weight, which was the highest dose tested [20].

No genotoxic activity was observed for l-theanine in the Salmonella mutagenicity assay, suggesting a general lack of carcinogenic potential [65]. Indeed, a recent systematic review concluded that l-theanine exerted multiple, diverse anticancer functions by inhibiting Akt/mTOR, ERK/NFkB, JAK2/STAT3, EGFR, VEGFR and Met pathways [13]. As with numerous other natural products and their associated pleiotropic activities, delineating cause-and-effect for l-theanine will require considerable additional work, especially in the clinical setting [92]. Given the recent surge of interest in l-theanine as a health supplement, further studies are warranted into the question of efficacy and transferability to the population at-large.

## 17. Conclusions

**Fig. 2 –:**
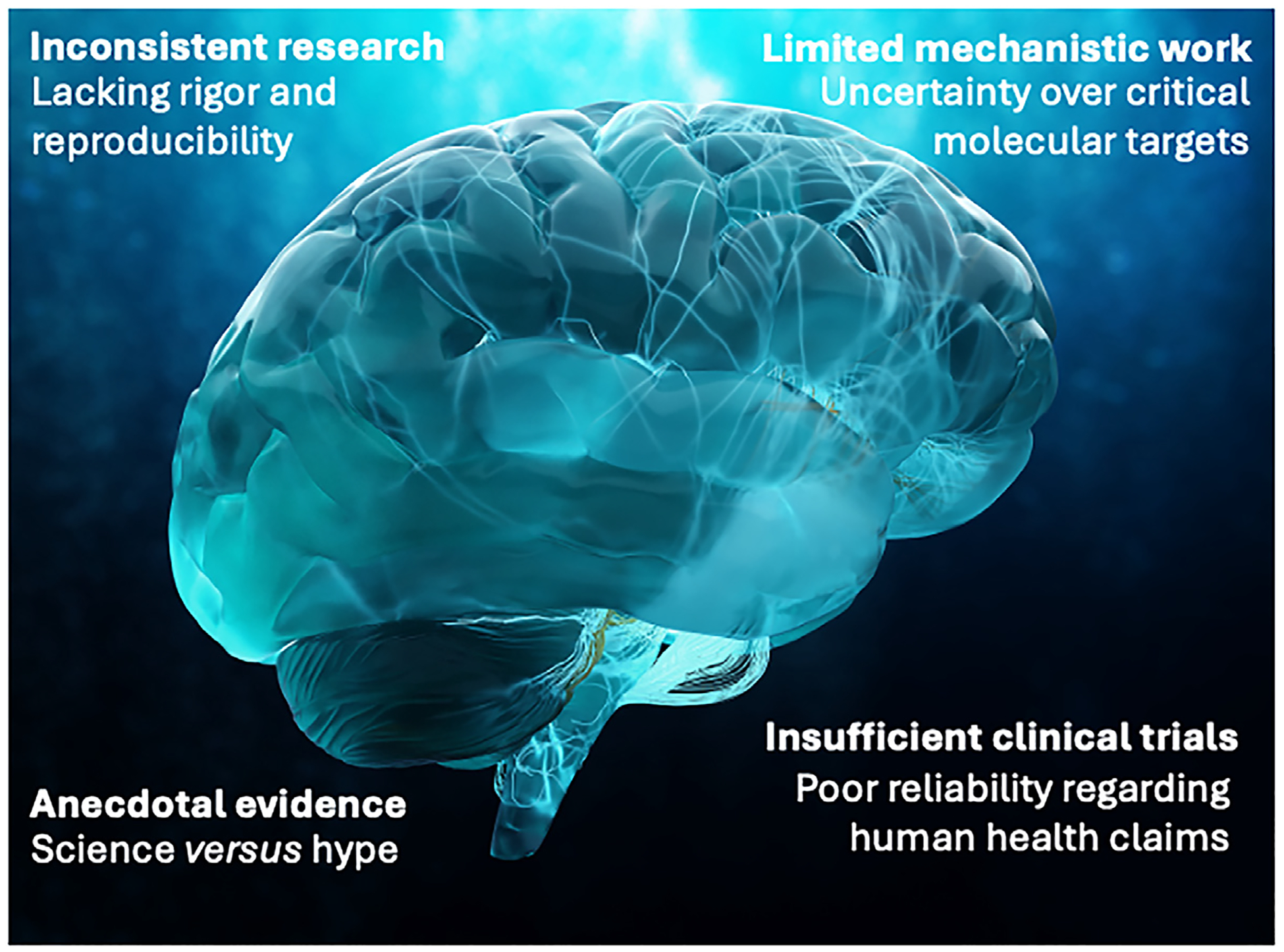
l-Theanine in brain health and relaxation.

Tea has been consumed for millennia, mainly for its stimulant properties due to caffeine. However, compared with coffee, moderate tea consumption appears to induce a state of wakefulness unaccompanied by agitation and other less desirable characteristics of caffeine intake. This physiological response has been attributed, in part, to tea catechins and l-theanine – an attribution that must be interpreted with caution [93], and that makes it critically important to distinguish l-theanine as a natural component of tea from its use as a nootropic nutraceutical at pharmacologic doses (Fig. 2). l-theanine is available in synthetic form and marketed as a natural nootropic and relaxant, with the suggestion of mild hypnotic and nootropic properties. l-Theanine has been studied toxicologically according to OECD international parameters and no adverse effects have been reported, even at high doses [94]. The bioavailability, kinetics, and metabolism of l-theanine are relatively well-established, from taking l-theanine in supplement form as capsules or as a natural component of green tea and its extracts.

Researchers have investigated the neuromodulatory effects of l-theanine in various clinical trials [95]. These are typically studies with small numbers of subjects and with measures of efficacy/ outcome that are difficult to verify in the absence of robust and validated biomarkers. The primary focus has been on improving cognitive function [53,84,86,96]. Collectively, the findings suggest some diminution of reaction time – hinting at increased cognitive function. Investigation into the reduction of stress and anxiety also yielded inconclusive results [41,52,97]. This ambiguity was mainly due to a lack of standardized methods and the small number of volunteers. There are, to date, no “gold standard” randomized, placebo-controlled trials examining the effects of l-theanine on sleep induction and maintenance, or indeed for many of the other attributes and benefits ascribed to l-theanine. It is also noteworthy that many studies on the biological properties of l-theanine come from one geographical area, and from one commercial/proprietary source of l-theanine, with data published in journals of moderate impact to a broad scientific community.

Finally, the general scientific guidance for stakeholders published in 2016 served as the basis for denial of a claim on theanine and attention [98]. We concur with this assessment and conclude that the available data on the biological properties of l-theanine are promising, but far too preliminary to unequivocally support many of the purported health claims [99]. l-Theanine as a major trending health supplement must be viewed in this context, and with appropriate caution when used in the wider population.

## Abbreviations:

ADHD: Attention Deficit/Hyperactivity Disorder
AMPA: α-amino-3-hydroxy-5-methyl-4-isoxazolepropionic acid
CB1: cannabinoid receptor 1
EGCG: epigallocatechin-3-gallate
GABA: g-aminobutyric acid
IUPHAR: The International Union of Basic and Clinical Pharmacology
MMSE: Mini-Mental State Examination
NMDA: N-methyl-D-aspartate
NOAEL: no observed adverse effect level
